# Associations between dietary patterns and cardiovascular disease risk in Canadian adults: a comparison of partial least squares, reduced rank regression and the simplified dietary pattern technique.

**DOI:** 10.23889/ijpds.v7i3.2103

**Published:** 2022-08-25

**Authors:** Svilena Lazarova, Mahsa Jessri

**Affiliations:** 1 Food, Nutrition and Health Program, University of British Columbia, Vancouver, British Columbia, Canada; 2 Centre for Health Services and Policy Research, University of British Columbia

## Objectives

We aimed to compare two data reduction techniques, partial least squares (PLS) and reduced rank regression (RRR), in identifying dietary patterns associated with a high cardiovascular disease (CVD) risk in Canadian adults, and to construct PLS- and RRR-based simplified dietary patterns as well as evaluating associations between derived patterns and CVD risk.

## Approach

Data were collected from 24-hour dietary recalls of adult respondents in two cycles of the nationally representative Canadian Community Health Survey (CCHS)-Nutrition: CCHS 2004 linked to health administrative databases (n = 12,313) and CCHS 2015 (n = 14,020). Using 39 food groups, PLS and RRR were applied for the identification of an energy-dense (ED), high-saturated-fat (HSF) and low-fiber-density (LFD) dietary pattern. Associations of derived dietary patterns with lifestyle characteristics and CVD incidence and mortality were examined using weighted multivariate regression and weighted multivariable-adjusted Cox-proportional hazard models, respectively. Random and systematic measurement errors were adjusted for in all statistical analyses.

## Results

PLS and RRR identified highly similar ED, HSF, LFD dietary patterns with common high positive loadings for fast food, carbonated drinks, salty snacks and solid fats, and high negative loadings for fruit, dark green vegetables, red and orange vegetables, other vegetables, whole grains, legumes and soy (≥|0.17|). Food groups with the highest loadings were summed to form simplified pattern scores. Although the dietary patterns were not significantly associated with CVD risk, they were positively associated with 402 kcal/d higher energy intake (P-trends <0.05) and higher obesity risk [PLS (OR: 2.09; 95% CI: 1.62, 2.7) and RRR (OR: 1.76; 95% CI: 1.44, 2.17)] (P-trends <0.0001) in the fourth quartiles as compared to the first.

## Conclusion

PLS and RRR were shown to be equally effective for derivation of a high-CVD-risk dietary pattern among Canadian adults. This research highlights the importance of leveraging linked data to inform public health nutrition policies. Further research is warranted on the role of major dietary components in cardiovascular health.

